# Impact of sleep quality and chronotype on self-reported erectile function in young adults presenting with erectile complaints: a prospective observational study

**DOI:** 10.1038/s41443-025-01089-4

**Published:** 2025-05-21

**Authors:** Ali Nebioğlu, Mert Başaranoğlu, Selahittin Çayan

**Affiliations:** 1 Department of Urology, Mersin City Training and Research Hospital, Mersin, Turkey; 2 Department of Urology, Mersin University Faculty of Medicine, Mersin, Turkey

**Keywords:** Sexual dysfunction, Erectile dysfunction

## Abstract

Erectile complaints are prevalent among young adults, with various factors potentially affecting sexual function and quality of life. This study investigates the impact of sleep quality and chronotype on erectile function in young adults who present with self-reported erectile difficulties. In this prospective study, 249 men with ED were included, assessed from January 2023 to July 2024. Sociodemographic and clinical data were collected for all participants, including age, body mass index (BMI), comorbid diseases, and hormonal parameters. All patients completed the following questionnaires: International Index of Erectile Function (IIEF-5), Pittsburgh Sleep Quality Index (PSQI), Morningness-Eveningness Questionnaire (MEQ), Depression Anxiety Stress Scales-42 (DASS-42), and General Health Questionnaire-28 (GHQ-28). Statistical analyses, including Spearman’s correlation and Kruskal-Wallis tests, evaluated the relationships between sleep quality, chronotype, and ED. The average age was 31.03 ± 8.06 years. Significant correlations were found: Poor sleep quality was linked to lower erectile function (r = −0.379, *p* < 0.001), while morning chronotype correlated with better erectile function (r = 0.424, *p* < 0.001). Regression analysis confirmed these as significant predictors of ED (R^2^ = 0.200, *p* < 0.001). Sleep quality and chronotype are crucial factors in ED management, suggesting targeted interventions could be beneficial.

## Introduction

Sexual health constitutes a crucial aspect of an individual’s overall health and quality of life [1]. According to the World Health Organization (WHO), sexual health encompasses not only physical well-being but also mental and social well-being [2]. In this context, sexual dysfunctions can significantly impact individuals’ psychological and social lives [3].

Erectile dysfunction (ED) is among the most prevalent sexual disorders in men and can significantly impair their quality of life. ED is defined as the inability to achieve or maintain a penile erection sufficient for satisfactory sexual intercourse [4]. Epidemiological studies indicate that the prevalence of ED is much higher than previously estimated [5]. Major cohort studies have provided significant insights into the epidemiology of ED. The Massachusetts Male Aging Study, a landmark community-based study, reported ED prevalence rates ranging from 30–52% [6]. Similarly, the European Male Ageing Study (EMAS), a large-scale multicenter population-based study investigating age-related hormonal changes and various health outcomes in men, demonstrated comparable prevalence rates and highlighted the significant impact of age-related factors on erectile function [7]. This comprehensive study, which included 3369 men aged 40–79 years from eight European centers, provided crucial data about the relationship between age, hormonal status, and erectile function. In Turkey, a population-based study detected ED in 33% of men over the age of 40 [8]. These data demonstrate that ED is a significant and frequently encountered health issue on a global scale, with prevalence rates being relatively consistent across different populations and geographical regions.

The etiology of ED is multifactorial, involving the interaction of various factors encompassing psychological, neurological, endocrine, and vascular systems [9, 10]. In recent years, the potential effects of sleep disorders and circadian rhythm changes on ED have begun to attract researchers’ attention.

Sleep is a process of critical importance for human physiology, and disruptions in sleep quality can increase the risk of ED through various mechanisms, such as hormonal imbalances, oxidative stress, and inflammation [11, 12]. Sleep disturbances can affect erectile function through multiple pathways: (1) disruption of the hypothalamic-pituitary-gonadal axis leading to reduced testosterone production [13], (2) increased oxidative stress and inflammation that may impair endothelial function [14], (3) dysregulation of autonomic nervous system affecting smooth muscle function [15], and (4) psychological effects including increased anxiety and stress levels [16]. The effect of biological rhythms (chronotypes) on ED is an area that has not yet been sufficiently explored. Chronotypes reflect individuals’ preferences in their sleep-wake cycles and are generally classified as morning type, evening type, and intermediate type [17]. It is known that chronotype differences are associated with psychological factors such as coping with stress, anxiety, and depression [18–20]. The fact that these factors are also related to ED suggests a potential link between chronotypes and ED [21].

A better understanding of the relationship between sleep quality, chronotype, and ED may contribute to the development of new strategies for the prevention and treatment of ED. There is a limited number of studies on this subject in the literature [14]. Therefore, the objective of this research is to investigate the impact of sleep quality and variations in chronotype on ED.

## Materials and methods

### Study population and data collection

A prospective analysis was conducted on data from 403 male patients who presented with self-reported complaints of erectile difficulties at the Urology Clinic of Mersin University between January 1, 2023, and July 1, 2024. The initial evaluation included a detailed medical and sexual history, physical examination, and assessment of morning erections through the Sexual Health Inventory for Men (SHIM) questionnaire.

Exclusion criteria encompassed patients outside the 18–45 age range, individuals with hormonal parameters outside normal reference values or those lacking these measurements, patients with abnormal penile duplex doppler ultrasound findings suggesting vascular ED, patients with comorbid diseases (including history of pelvic or urological surgery, neurological disorders, or veno-occlusive dysfunction), patients with obstructive sleep apnea or using continuous positive airway pressure devices, regular tobacco and/or alcohol users, and those who did not provide informed consent.

To establish the psychogenic nature of ED, we employed a comprehensive diagnostic approach that included several key components. We first confirmed normal morning and nocturnal erections through patient history and SHIM questionnaire assessment. Physical examination findings were required to be normal, along with verification of a normal hormonal profile. Additionally, penile duplex Doppler ultrasound findings needed to demonstrate normal parameters, specifically a peak systolic velocity greater than 35 cm/s, end-diastolic velocity less than 5 cm/s, and resistance index above 0.9. Finally, we confirmed the absence of any organic risk factors or comorbidities that could contribute to ED symptoms.

Sociodemographic and clinical data were collected for all participants, including age, weight, height, body mass index (BMI), regular tobacco use, regular alcohol consumption, comorbid diseases, and hormonal parameters. All patients completed the following questionnaires: International Index of Erectile Function (IIEF-5), Pittsburgh Sleep Quality Index (PSQI), Morningness-Eveningness Questionnaire (MEQ), Depression Anxiety Stress Scales-42 (DASS-42), and General Health Questionnaire-28 (GHQ-28).

Ethical approval for this research was obtained from the Local Ethics Committee of Mersin University Faculty of Medicine, with the decision number 2024/801 dated September 4, 2024.

### Data collection and quality control

To ensure data completeness and quality, we implemented a rigorous data collection protocol. All questionnaires were administered under direct supervision of trained research staff during clinic visits, allowing immediate identification and addressing of missing or unclear responses. Each questionnaire was reviewed for completeness before the participant left the clinic, and any missing responses were immediately addressed. For participants who initially missed clinic visits, we implemented a follow-up protocol including phone calls and rescheduling options. Data verification was performed through double data entry by two independent researchers, with any discrepancies resolved through consensus and reference to original forms. Regular data quality checks were conducted throughout the study period. As a result of these procedures, we achieved complete data for all 249 participants included in the final analysis.

### Measurements

#### International index of erectile function (IIEF-5)

The assessment form consists of five questions and is an internationally validated tool used for diagnosing ED and evaluating treatment efficacy [24, 25]. The Turkish adaptation of the scale has undergone the necessary psychometric analyses, and its reliability and validity studies have been completed [26].

#### Pittsburgh sleep quality index (PSQI)

The scale is a nineteen-item questionnaire designed to evaluate individuals’ sleep habits and provide a subjective analysis of sleep quality over the past month [27]. The scale comprises seven main components: subjective sleep quality, sleep latency, habitual sleep efficiency, sleep duration, use of hypnotic medication, sleep disturbances, and daytime dysfunction. Each component is rated on a scale from zero to three, and the sum of the scores constitutes the global score. The global score ranges from zero to twenty-one, with scores above five indicating poor sleep quality. The Turkish version has been validated [28].

#### Morningness-eveningness questionnaire (MEQ)

The assessment instrument consists of nineteen items, comprising five open-ended questions and fourteen multiple-choice items [29]. Participants indicate their preferred sleep-wake cycle patterns and optimal periods for physical and mental performance. The total score ranges from sixteen (extreme evening chronotype) to eighty-six (extreme morning chronotype). According to the scale’s cut-off points, total scores above fifty-eight indicate a morning chronotype, while scores below forty-two indicate an evening chronotype. The Turkish version has been validated through a comprehensive process including translation and back-translation, demonstrating high internal consistency (Cronbach’s alpha = 0.84), excellent test-retest reliability (r = 0.88, *p* < 0.001), strong construct validity through factor analysis, and concurrent validity with sleep logs and actigraphy data [30].

#### Depression anxiety stress scales-42 (DASS-42)

The DASS-42 is a forty-two-item psychometric assessment instrument designed to evaluate symptoms of depression, anxiety, and stress experienced during the preceding week [31]. Each subscale consists of fourteen items rated from zero to three. The possible score range for each subscale varies between zero and forty-two. The Turkish version has demonstrated good psychometric properties [32].

#### General health questionnaire-28 (GHQ-28)

GHQ-28 is a psychometric assessment instrument comprising twenty-eight items [33]. The conventional scoring methodology assigns zero points to the first and second options and one point to the third and fourth options. The Turkish version has been validated with a cut-off point of five points [34].

#### Statistical analysis

Statistical analyses in this study were conducted using IBM SPSS Statistics (version 25.0, IBM Corp., Armonk, NY, USA). The significance level was set at *p* < 0.05. All statistical tests were performed as two-sided tests. A total of 249 participants were included in the study. Mean, standard deviation, median, and interquartile range were calculated for the PSQI, IIEF-5, and MEQ scores. The Shapiro-Wilk test was used to assess the normality of data distribution. Test results indicated that PSQI (W = 0.9304, *p* < 0.001), IIEF-5 (W = 0.9077, *p* < 0.001), and MEQ (W = 0.8984, *p* < 0.001) scores did not follow a normal distribution.

To evaluate the relationship between sleep quality (PSQI) and ED (IIEF-5), Spearman’s rank correlation coefficient was employed due to the non-normal distribution of data. To assess the relationship between chronotypes and ED, participants were categorized into three groups based on their MEQ scores: morning type (59 and above), intermediate type (42–58), and evening type (41 and below). The non-parametric Kruskal-Wallis test was applied to determine if there were differences in IIEF-5 scores among chronotypes. Dunn’s test was used for post-hoc analyses, with Bonferroni correction applied.

Multiple linear regression analysis was performed to evaluate the independent effects of sleep quality and chronotypes on ED. IIEF-5 score was included in the model as the dependent variable, while the PSQI score and chronotypes were included as independent variables. The model was adjusted for potential confounding factors such as age, BMI, smoking status, and comorbid diseases.

Sample size calculation was performed prior to the study using G*Power (version 3.1.9.4, Universität Düsseldorf, Germany). Based on previous studies investigating sleep quality and erectile function, we anticipated a medium effect size (f = 0.25). With α = 0.05 and power (1-β) = 0.80, the minimum required sample size was calculated as 159 participants. To account for potential dropouts and incomplete data, we aimed to recruit at least 200 participants.

All statistical analyses and graphical representations were performed in accordance with the principles of reproducibility and transparency.

## Results

The study included 249 participants. The demographic and clinical characteristics of the study population are presented in Table 1. The mean age was 31.03 ± 8.06 years (range: 18–45 years). The mean BMI was 24.8 ± 3.2 kg/m^2^ (range: 18.5–29.9). The majority of participants had completed higher education (68.3%) and were married (71.5%).

**Table 1:** Sociodemographic, clinical, and psychometric characteristics of study participants (*N* = 249).

Characteristic	Value
Sociodemographic Characteristics	
Age (years), mean ± SD	31.03 ± 8.06
Age range	18–45
BMI (kg/m²), mean ± SD	24.8 ± 3.2
Education Level, n (%)	
High School	72 (28.9)
University	147 (59.0)
Postgraduate	30 (12.1)
Marital Status, n (%)	
Single	142 (57.0)
Married	98 (39.4)
Divorced	9 (3.6)
Monthly Income Level, n (%)	
Low (<2× minimum wage)	58 (23.3)
Middle (2–4× minimum wage)	143 (57.4)
High (>4× minimum wage)	48 (19.3)
Psychometric Assessment Scores	
IIEF-5 score, mean ± SD	12.67 ± 5.85
PSQI score, mean ± SD	5.64 ± 3.50
MEQ score, mean ± SD	55.13 ± 21.77
Chronotype Distribution	
Morning Type, n (%)	154 (61.8)
Intermediate Type, n (%)	6 (2.4)
Evening Type, n (%)	89 (35.7)
DASS-42 Subscales	
Depression, mean ± SD	8.45 ± 4.32
Anxiety, mean ± SD	7.23 ± 3.98
Stress, mean ± SD	12.67 ± 5.12
GHQ-28	
Score ≥5, n (%)	82 (32.9)
Score <5, n (%)	167 (67.1)

*SD* standard deviation, *BMI* body mass index, *IIEF-5* international index of erectile function-5 (range: 5–25, higher scores indicate better function), *PSQI* pittsburgh sleep quality index (range: 0–21, higher scores indicate worse sleep quality), *MEQ* morningness-eveningness questionnaire (range: 16–86, higher scores indicate morning preference), *DASS-42* depression anxiety stress scales-42, *GHQ-28* general health questionnaire-28.

Hormonal parameters were measured between 8:00 and 10:00 AM after overnight fasting to minimize diurnal variation effects. The mean values (± SD) and ranges were as follows: total testosterone 4.85 ± 1.12 ng/mL (2.49–8.36), free testosterone 15.2 ± 3.8 pg/mL (9.3–26.5), FSH 4.2 ± 1.8 mIU/mL (1.5–12.4), LH 4.8 ± 1.6 mIU/mL (1.7–8.6), prolactin 8.6 ± 3.2 ng/mL (4.1–15.2), and estradiol 25.4 ± 8.6 pg/mL (11.3–39.8). All values were within their respective reference ranges, though we acknowledge that single time-point measurements may not fully capture the dynamic nature of hormonal regulation.

Clinical assessment revealed varying degrees of erectile dysfunction based on IIEF-5 scores: severe ED (18.1%), moderate ED (24.9%), mild to moderate ED (29.3%), mild ED (18.9%), and no ED (8.8%). The mean PSQI global score was 5.64 ± 3.50 (range: 0–11). Based on MEQ scores, participants were classified as morning type (*n* = 154, 61.8%), intermediate type (*n* = 6, 2.4%), and evening type (*n* = 89, 35.8%). DASS-42 subscale analysis showed mean scores of 8.45 ± 4.32 for depression, 7.23 ± 3.98 for anxiety, and 12.67 ± 5.12 for stress. The mean GHQ-28 score was 4.32 ± 2.87, with 82 participants (32.9%) scoring above the clinical cut-off point of 5.

**Fig. 1:**
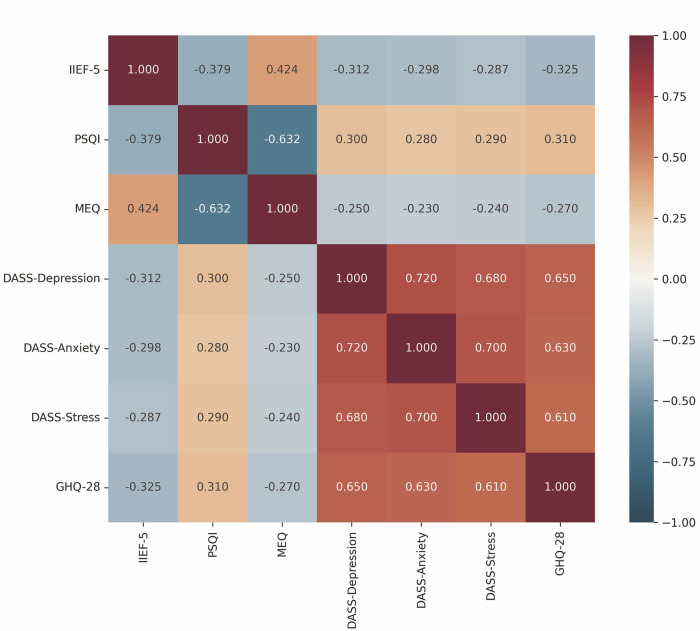
Correlation matrix heatmap of study variables. Figure displays a color-coded correlation matrix of all study variables, including IIEF-5, PSQI, MEQ, DASS-42 subscales (Depression, Anxiety, Stress), and GHQ-28 scores. The heatmap uses a red-blue color gradient (red for negative correlations, blue for positive correlations) with correlation coefficients displayed in each cell. Key relationships highlighted include: negative correlation between IIEF-5 and PSQI (r = −0.379), positive correlation between IIEF-5 and MEQ (r = 0.424), and strong negative correlation between PSQI and MEQ (r = −0.632). All psychological parameters (DASS-42 subscales and GHQ-28) show significant negative correlations with erectile function (IIEF-5) and morning preference (MEQ), while positively correlating with poor sleep quality (PSQI). All correlations significant at *p* < 0.001. IEF-5 international index of erectile function-5, PSQI pittsburgh sleep quality index, MEQ morningness-eveningness questionnaire, DASS depression anxiety stress scales-42, GHQ-28 general health questionnaire-28.

A significant negative correlation was observed between PSQI and IIEF-5 scores (r = −0.379, *p* < 0.001), indicating that poorer sleep quality is associated with lower erectile function. MEQ scores showed a positive correlation with IIEF-5 scores (r = 0.424, *p* < 0.001), suggesting that morning-type individuals tend to have better erectile function. A strong negative correlation was found between PSQI and MEQ scores (r = −0.632, *p* < 0.001), indicating that evening-type individuals generally report poorer sleep quality (Table 2, Fig. 1).

**Table 2:** Correlation matrix of pittsburgh sleep quality index (PSQI), international index of erectile function-5 (IIEF-5), and morningness-eveningness questionnaire (MEQ) scores.

Variable	PSQI	IIEF-5	MEQ
PSQI	1	−0.379243433	−0.6318781485
IIEF-5	−0.379243433	1	0.4239814495
MEQ	−0.6318781485	0.4239814495	1

Values represent Spearman’s correlation coefficients. All correlations are significant at *p* < 0.001.

*PSQI* pittsburgh sleep quality index (higher scores indicate worse sleep quality), *IIEF-5* international index of erectile function-5 (higher scores indicate better erectile function), *MEQ* morningness-eveningness questionnaire (higher scores indicate morning chronotype).

Further analysis revealed significant correlations between psychological parameters and erectile function. DASS-42 depression scores showed a moderate negative correlation with IIEF-5 scores (r = −0.312, *p* < 0.001), as did anxiety scores (r = −0.298, *p* < 0.001) and stress scores (r = −0.287, *p* < 0.001). Similarly, higher GHQ-28 scores were associated with lower IIEF-5 scores (r = −0.325, *p* < 0.001).

Multiple linear regression analysis revealed that both PSQI and MEQ scores were significant predictors of IIEF-5 scores (Table 3). The regression model explained 20% of the variance in IIEF-5 scores (R² = 0.200, F (2, 246) = 30.83, *p* < 0.001). PSQI scores negatively predicted IIEF-5 scores (β = −0.310, *p* = 0.012), while MEQ scores were positive predictors (β = 0.083, *p* < 0.001).

**Table 3:** Multiple regression analysis of pittsburgh sleep quality index (PSQI) and morningness-eveningness questionnaire (MEQ) scores on international index of erectile function-5 (IIEF-5) scores.

Variable	Coefficient	Std. Error	t-statistic	P>|t|	[0.025	0.975]
Constant	9.8724	1.655	5.965	0.000	6.612	13.132
PSQI	−0.3102	0.123	−2.520	0.012	−0.553	−0.068
MEQ	0.0825	0.020	4.172	0.000	0.044	0.121

Model R^2^ = 0.200, F(2, 246) = 30.83, *p* < 0.001. Confidence intervals are shown at 95% level. The model was adjusted for age, BMI, smoking status, and comorbid diseases.

*PSQI* pittsburgh sleep quality index, *IIEF-5* international index of erectile function-5, *MEQ* morningness-eveningness questionnaire.

Participants were divided into three chronotypes based on MEQ scores: Evening Type (*n* = 89), Intermediate Type (*n* = 6), and Morning Type (*n* = 154). One-way ANOVA revealed a significant difference in IIEF-5 scores among chronotypes (F (2, 246) = 40.29, *p* < 0.001) (Table 4). Post-hoc analysis using Tukey’ s HSD test showed that Morning Type individuals had significantly higher IIEF-5 scores compared to Evening Type individuals (mean difference = 6.09, *p* < 0.001) (Table 5).

**Table 4:** One-way ANOVA comparison of international index of erectile function-5 (IIEF-5) scores between chronotypes.

Source	Sum of Squares	df	F	Pr(>F)
C(Chronotype)	2096.62	2	40.2921966816	7.304334347e–16
Residual	6400.37	246		

Chronotypes were categorized based on MEQ scores: Evening Type (≤41), Intermediate Type (42-58), and Morning Type (≥59).

*IIEF-5* international index of erectile function-5, *df* degrees of freedom, *F* F-statistic; *Pr(>F)* p-value.

**Table 5:** Post-hoc analysis: Tukey HSD multiple comparison of IIEF-5 scores between chronotypes.

Group 1	Group 2	Mean Difference	p-adjusted	Lower	Upper	Reject
Evening Type	Intermediate Type	3.0019	0.3449	−2.0712	8.0749	False
Evening Type	Morning Type	6.0906	0.0	4.4891	7.6921	True
Intermediate Type	Morning Type	3.0887	0.3144	−1.9162	8.0937	False

Mean Difference: difference in IIEF-5 scores between groups; p-adjusted: Bonferroni-adjusted p-value; Lower/Upper: 95% confidence interval bounds; Reject: whether the null hypothesis of no difference is rejected at α = 0.05. Evening Type (*n* = 89), Intermediate Type (*n* = 6), Morning Type (*n* = 154).

*IIEF-5* international index of erectile function-5.

Among the 249 participants included in the final analysis, IIEF-5 scores showed varying degrees of erectile function:

- Severe ED (IIEF-5: 5–7): 45 patients (18.1%)
- Moderate ED (IIEF-5: 8–11): 62 patients (24.9%)
- Mild to Moderate ED (IIEF-5: 12–16): 73 patients (29.3%)
- Mild ED (IIEF-5: 17–21): 47 patients (18.9%)
- No ED (IIEF-5: 22–25): 22 patients (8.8%)

This distribution highlights an important finding: while all participants initially presented with self-reported erectile complaints, objective IIEF-5 scores revealed varying degrees of erectile function, including a subset of patients with scores in the normal range.

## Discussion

This study aimed to investigate the relationship between sleep quality, chronotype, and ED. Our findings reveal significant associations between these parameters and provide insights into potential underlying mechanisms. The psychological and physiological implications of these relationships extend beyond individual symptoms, suggesting broader impacts on men’s health and quality of life.

The relationship between sleep quality and erectile function appears to operate through several interconnected pathways. Our findings of a significant negative correlation between PSQI and IIEF-5 scores (r = −0.379, *p* < 0.001) align with current understanding of sleep’s role in neuroendocrine regulation. Sleep disruption can affect erectile function through multiple mechanisms:

First, the Hypothalamic-Pituitary-Gonadal (HPG) axis, crucial for testosterone production and regulation, is highly sensitive to sleep patterns. While our participants had normal hormone levels, the dynamic nature of these interactions suggests that even subtle disruptions in sleep patterns might influence erectile function through altered hormonal secretion patterns and receptor sensitivity.

Second, poor sleep quality leads to autonomic nervous system dysregulation, particularly affecting the balance between sympathetic and parasympathetic activity. This imbalance can directly impact erectile function through altered vascular responses and smooth muscle tone.

Third, the relationship between chronotype and erectile function, demonstrated by the positive correlation between MEQ and IIEF-5 scores (r = 0.424, *p* < 0.001), suggests a role for circadian rhythm alignment. Morning-type individuals may benefit from better synchronization between their biological rhythms and societal schedules, potentially leading to more stable sleep patterns and optimal hormonal profiles.

The selection of a relatively young age group (18–45 years) in our study was deliberate and based on several key methodological considerations. First, by focusing on younger patients, we aimed to minimize the confounding effects of age-related comorbidities and organic causes of ED, which become increasingly prevalent with age. This approach allowed us to better isolate and examine the specific relationships between sleep patterns, chronotype, and erectile function. Second, young adults typically demonstrate more pronounced variations in sleep patterns and chronotype preferences compared to older populations, making them an ideal group for investigating these relationships. Third, the psychological component of ED is particularly relevant in younger populations, where organic causes are less prevalent, allowing us to better examine the interplay between sleep patterns, psychological factors, and erectile function.

However, we acknowledge that this age-specific focus represents a limitation in terms of generalizability. Our findings may not directly apply to older populations, where ED often presents with multiple contributing factors including vascular, neurological, or systemic health conditions. The interaction between sleep quality, chronotype, and erectile function may differ significantly in older age groups due to these comorbidities. Future research should investigate these relationships across different age groups to better understand how age-related factors interact with sleep patterns and chronotype in affecting erectile function. Such studies would provide valuable insights into whether the associations we observed in young adults persist or change with age, and how they might be modified by age-related health conditions.

While all participants had hormone levels (Total testosterone, LH, FSH, Estradiol, and Prolactin) within normal reference ranges as per our inclusion criteria, we acknowledge that single-point measurements may not fully capture the dynamic nature of hormonal regulation. Hormonal parameters, particularly testosterone, exhibit diurnal variations and can be influenced by sleep-wake patterns. Although we did not include the specific hormone values in our results, as they were all within normal ranges, future studies might benefit from multiple time-point measurements to better understand the relationship between circadian hormonal variations, sleep patterns, and erectile function.

Our study gains additional significance as it is the first comprehensive study in the literature comparing the effects of sleep quality and chronotypes on erectile function using this combination of validated assessment tools.

**Fig. 2:**
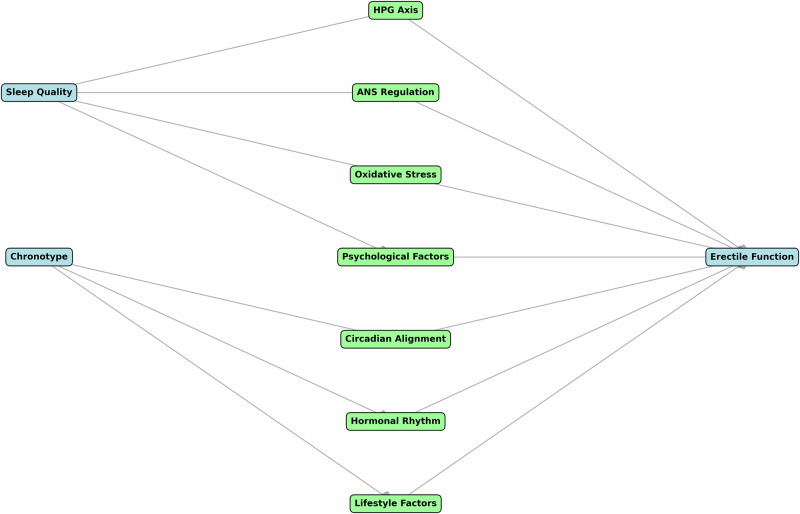
Proposed mechanistic pathways linking sleep quality, chronotype, and erectile function. Figure illustrates a directed graph showing the relationships between sleep quality, chronotype, and erectile function. The diagram depicts multiple pathways: Sleep quality influences erectile function through HPG axis regulation, autonomic nervous system regulation, oxidative stress, and psychological factors. Chronotype affects erectile function through circadian alignment, hormonal rhythm, and lifestyle factors. All pathways converge on erectile function, with arrows indicating directional relationships. Morning chronotype is associated with better sleep quality and improved parameters across all pathways, ultimately contributing to better erectile function. The diagram was generated using a computational approach to visualize the complex interrelationships between these factors based on current evidence in the literature.

Our findings align with and extend the recent work by Gurel et al. [22], who also found that poor sleep quality and evening chronotype are significant risk factors for ED. However, our study provides several novel contributions beyond confirming these associations. First, we included comprehensive psychological assessments using DASS-42 and GHQ-28, revealing specific correlations between chronotype, psychological parameters, and ED severity. Second, our rigorous diagnostic approach, including penile Doppler ultrasound and standardized hormonal profiling, helps establish more definitive causal relationships. Third, our focus on young adults with self-reported complaints, rather than just IIEF-5 scores, revealed important discrepancies between subjective complaints and objective measures. Finally, our detailed mechanistic analysis and visual representation (Fig. 2) provide new insights into the physiological pathways linking sleep patterns and erectile function.

Recent studies have further strengthened the connection between sleep disorders and ED. Zhang et al. [14] demonstrated that chronic sleep restriction significantly impacts endothelial function and nitric oxide production, key factors in erectile function. Additionally, Li et al. [23] reported that circadian rhythm disruption affects not only testosterone levels but also androgen receptor sensitivity, potentially explaining the mechanism behind chronotype-related differences in erectile function. These findings support our observations regarding the relationship between chronotype, sleep quality, and erectile function.

To better illustrate these complex relationships, we have provided a comprehensive mechanistic diagram (Fig. 2) that depicts the potential pathways through which sleep quality and chronotype may influence erectile function. The figure demonstrates how sleep disruption can affect erectile function through multiple mechanisms: (1) HPG axis disruption leading to altered hormonal profiles, (2) autonomic nervous system imbalance affecting vascular response, (3) increased oxidative stress and inflammation impacting endothelial function, and (4) psychological factors including anxiety and depression. Similarly, the figure illustrates how chronotype influences erectile function through: (1) circadian alignment with physiological processes, (2) synchronization of hormonal rhythms, particularly testosterone, (3) lifestyle and behavioral patterns associated with different chronotypes, and (4) variation in stress response patterns between morning and evening types.

In conclusion, our findings underscore the potential importance of sleep quality and chronotype on erectile function. Interventions targeting sleep quality and considering individual circadian preferences (such as sleep hygiene education, cognitive-behavioral therapies, pharmacological treatments, or early sexual activity for morning-type individuals) may be beneficial in the management of ED. Future research should further elucidate these relationships using larger samples and objective sleep and circadian rhythm measurements, employing longitudinal designs. Additionally, investigating the underlying physiological mechanisms, such as hormonal changes associated with sleep patterns and chronotypes, could provide clinicians with valuable insights for developing targeted interventions for ED.

## Data Availability

The data that support the findings of this study are available on request from the corresponding author. The data are not publicly available due to their containing information that could compromise the privacy of research participants.
